# The Advertisements of Religious Newspapers

**Published:** 1887-07

**Authors:** 


### The Advertisements of Religious Newspapers.

No thoughtful physician has examined the advertisements of the religious press without a feeling of shame. Wickedness of every sort can there be found directly stated, or implied by crafty suggestion. A short time since we decided that a certain religious paper was unfit to be read by the family, and, as a reason for discontinuing the same, cut out the objectionable advertisements and with a note sent them to the publisher of the paper, asking that the same be no longer sent to us. After the bad advertisements were cut from the paper there was little left.

The publisher made a courteous reply. He confessed that he was ashamed of his advertisements, because they were in bad taste. We had objected to them because they were false, vicious, and evil. He said that he would discontinue all his contracts if it were possible. But he had ungardedly signed a contract with an advertising firm for all his advertising pages. He reserved the right to reject any advertisement unfit for the columns of a religious newspaper. But he said that when he called the agent's attention to these advertisements, insisting that they were unfit for the columns of his paper, he was met with the reply that the same advertisements were published in all the religious press; hence it was evident that they were suitable for this style of paper. An examination of the several religious papers published in this country convinced him that the agent was right. Hence he was compelled to continue the objectionable advertisements during the time of the contract.

It will be seen that this gentleman only objected to the advertisements because he regarded them as in bad taste. He even said that some of his friends highly regarded some of these medicines. He was quite shocked at our claim that these were false in all relations and unsafe to have in any decent family; that they tended to neutralize whatever good the reading pages might contain. But the publishers of these journals are after the almighty dollar, and are willing to advertise the devil in the families of the people if they can only secure some of the devil's money.

Frankly, we believe that many of these gentlemen are so ignorant in all matters pertaining to medicine that they are unable to comprehend the claim of the medical profession that all advertisements pertaining to quackery are evil, and that continually. How shall they be educated? Perhaps personal instruction by some competent member of the profession would accomplish much. For the rest, we shall be compelled to await the gradual dissemination of the truth.

Meantime those who have families of children will take care that they are not corrupted by the viciousness of the advertising columns of the religious press.--*American Lancet.*

